# Supplementary material for: Risk factors for lactation mastitis in China: A systematic review and meta-analysis
Source: PLoS One. 2021 May 13;16(5):e0251182. doi: 10.1371/journal.pone.0251182 (PMC8118550; doi:10.1371/journal.pone.0251182)
Supplement: S2 File — (PDF) [file pone.0251182.s002.pdf]

National Institute for Health Research

UNIVERSITY of York
Centre for Reviews and Dissemination

Systematic review

1. * Review title.

Give the title of the review in English

The risk factors association with lactation mastitis in China: A systematic review and Meta-analysis

2. Original language title.

For reviews in languages other than English, give the title in the original language. This will be displayed with the English language title.

3. * Anticipated or actual start date.

Give the date the systematic review started or is expected to start.

15/04/2020

4. * Anticipated completion date.

Give the date by which the review is expected to be completed.

15/07/2020

5. * Stage of review at time of this submission.

Tick the boxes to show which review tasks have been started and which have been completed. Update this field each time any amendments are made to a published record.

Reviews that have started data extraction (at the time of initial submission) are not eligible for inclusion in PROSPERO. If there is later evidence that incorrect status and/or completion date has been supplied, the published PROSPERO record will be marked as retracted.

This field uses answers to initial screening questions. It cannot be edited until after registration.

The review has not yet started: No

Review stage	Started	Completed
Preliminary searches	Yes	No
Piloting of the study selection process	No	No
Formal screening of search results against eligibility criteria	No	No
Data extraction	No	No
Risk of bias (quality) assessment	No	No
Data analysis	No	No

Provide any other relevant information about the stage of the review here.

6. * Named contact.

The named contact is the guarantor for the accuracy of the information in the register record. This may be any member of the review team.

baoyong lai

Email salutation (e.g. "Dr Smith" or "Joanne") for correspondence:

Dr lai

7. * Named contact email.

Give the electronic email address of the named contact.

by_lai@126.com

8. Named contact address

Give the full institutional/organisational postal address for the named contact.

Beijing University of Chinese Medicine

9. Named contact phone number.

Give the telephone number for the named contact, including international dialling code.

15901286096

10. * Organisational affiliation of the review.

Full title of the organisational affiliations for this review and website address if available. This field may be completed as 'None' if the review is not affiliated to any organisation.

The third affiliated hospital of Beijing University of Chinese Medicine

Organisation web address:

11. * Review team members and their organisational affiliations.

Give the personal details and the organisational affiliations of each member of the review team. Affiliation refers to groups or organisations to which review team members belong. **NOTE: email and country now MUST be entered for each person, unless you are amending a published record.**

Dr baoyong lai. The third affiliated hospital of Beijing University of Chinese Medicine
liyan Jia. The school of traditional Chinese Medicine, Beijing University of Chinese Medicine
Dr Ai-Jing CHu. The third affiliated hospital of Beijing University of Chinese Medicine
Dr Shibing Liang. Centre for Evidence-Based Chinese Medicine, Beijing University of Chinese Medicine
Professor Xiaohua Pei. The third affiliated hospital of Beijing University of Chinese Medicine

12. * Funding sources/sponsors.

Details of the individuals, organizations, groups, companies or other legal entities who have funded or sponsored the review.

none

Grant number(s)

State the funder, grant or award number and the date of award

13. * Conflicts of interest.

List actual or perceived conflicts of interest (financial or academic).

None

14. Collaborators.

Give the name and affiliation of any individuals or organisations who are working on the review but who are not listed as review team members. **NOTE: email and country must be completed for each person, unless you are amending a published record.**

15. * Review question.

State the review question(s) clearly and precisely. It may be appropriate to break very broad questions down into a series of related more specific questions. Questions may be framed or refined using PI(E)COS or similar where relevant.

1. What risk factors are significantly associated with lactation mastitis in Chinese women?

To systemically evaluate the risk factors associated with the lactation mastitis in China. The doctors and lactation mothers can identify the risk factors of lactation mastitis, which is beneficial to reduce the incidence rate of lactation mastitis and improve breastfeeding rate in Asia.

16. * Searches.

State the sources that will be searched (e.g. Medline). Give the search dates, and any restrictions (e.g. language or publication date). Do NOT enter the full search strategy (it may be provided as a link or attachment below.)

We will search PubMed, Web of Science, Chinese Biomedical Literature Database (Sinomed), China National Knowledge Infrastructure (CNKI), Wanfang Database and China Science Technology Journal Database (VIP) from their inception to May 1st, 2020?update according to the actual searching?. English and Chinese language publications were included.

17. URL to search strategy.

Upload a file with your search strategy, or an example of a search strategy for a specific database, (including the keywords) in pdf or word format. In doing so you are consenting to the file being made publicly accessible. Or provide a URL or link to the strategy. Do NOT provide links to your search **results**.

Alternatively, upload your search strategy to CRD in pdf format. Please note that by doing so you are consenting to the file being made publicly accessible.

Do not make this file publicly available until the review is complete

18. * Condition or domain being studied.

Give a short description of the disease, condition or healthcare domain being studied in your systematic review.

Lactation mastitis (LM) is one of the most common diseases in puerperal women. It is clinically characterized

by red, swollen, hot and the tender of breast, and the patient usually is accompanied by high fever, headache, and other influenza-like symptoms. The incidence of LM varied widely across populations, variations in breastfeeding methods and other factors. Up to approximately 3–24% of breastfeeding mothers could suffer LM in their lifetime. Breastfeeding was reported to have the beneficial influence on the immediate and long term infant and maternal health outcomes.

However, it was of concern that the previous survey in china reported the breastfeeding rate of infants aged 1-2 months was 59.4% to 66.5%. The main reasons directly leading to failure of breastfeeding were LM and the discomfort associated with the condition. It is therefore of great significance to explore the risk factors association with LM and to extend the overall duration of breastfeeding. We focused on the risk factors association with lactation mastitis in China in this research.

19. * Participants/population.

Specify the participants or populations being studied in the review. The preferred format includes details of both inclusion and exclusion criteria.

The exposure or case group was the participants with lactation mastitis.

20. * Intervention(s), exposure(s).

Give full and clear descriptions or definitions of the interventions or the exposures to be reviewed. The preferred format includes details of both inclusion and exclusion criteria.

Intervention or exposure was any risk factor association with lactation mastitis.

21. * Comparator(s)/control.

Where relevant, give details of the alternatives against which the intervention/exposure will be compared (e.g. another intervention or a non-exposed control group). The preferred format includes details of both inclusion and exclusion criteria.

The control group was the lactation mother without mastitis or the healthy women who had lactation experience.

22. * Types of study to be included.

Give details of the study designs (e.g. RCT) that are eligible for inclusion in the review. The preferred format includes both inclusion and exclusion criteria. If there are no restrictions on the types of study, this should be stated.

We included cohort study and case-control study enrolling any risk factors association with LM in Chinese women. Eligible studies must include at least 30 women in each group, regardless of their age or race.

23. Context.

Give summary details of the setting or other relevant characteristics, which help define the inclusion or exclusion criteria.

24. * Main outcome(s).

Give the pre-specified main (most important) outcomes of the review, including details of how the outcome is defined and measured and when these measurement are made, if these are part of the review inclusion criteria.

The data will be extracted to generate the odds ratios (with confidence limits) for effect of the risk factors.
risk factors associated with the lactation mastitis patients in China.

* Measures of effect

Please specify the effect measure(s) for you main outcome(s) e.g. relative risks, odds ratios, risk difference, and/or 'number needed to treat.

The data was presented by odds ratios (OR) with 95% confidence interval (CI).
Variables with different units will be converted to a uniform unit.

25. * Additional outcome(s).

List the pre-specified additional outcomes of the review, with a similar level of detail to that required for main outcomes. Where there are no additional outcomes please state ‘None’ or ‘Not applicable’ as appropriate to the review

Not applicable

* Measures of effect

Please specify the effect measure(s) for you additional outcome(s) e.g. relative risks, odds ratios, risk difference, and/or 'number needed to treat.

none

26. * Data extraction (selection and coding).

Describe how studies will be selected for inclusion. State what data will be extracted or obtained. State how this will be done and recorded.

Two authors were independently selected the studies and extracted the detailed data of the eligible trials. The items for data extraction were first authors and year of publication, the detailed information of methodology, characteristics of participants, sample size, descriptions of risk factors association with LM, the incidence of LM or incident cases of LM, etc. Any discrepancies regarding study selection and data extraction was resolved through consensus and arbitrated by the third author if necessary.

27. * Risk of bias (quality) assessment.

State which characteristics of the studies will be assessed and/or any formal risk of bias/quality assessment tools that will be used.

We evaluated the qualities of all included studies which trial types were case-control studies and cohort studies separately according to the criteria of Newcastle-Ottawa Scale (NOS). The “star” scoring system was used to evaluate the methodological quality of the included studies. A star was described as an appropriate entry, with each star representing one score. The possible NOS assessment score ranged from zero to nine points. The study with a score higher than seven stars was considered to be of high quality. The study with a score equal to seven stars was considered to be of medium quality. The study with a score equal to or less than six stars was categorized into low-quality. Any disagreements will be resolved by discussion with a third author.

28. * Strategy for data synthesis.

Describe the methods you plan to use to synthesise data. This **must not be generic text** but should be **specific to your review** and describe how the proposed approach will be applied to your data. If meta-analysis is planned, describe the models to be used, methods to explore statistical heterogeneity, and software package to be used.

We used RevMan Software 5.3 from Cochrane collaboration to perform statistical analyses. The dichotomous data was presented by odds ratios (OR) with 95% confidence interval (CI). We assessed statistical heterogeneity by using the I^2 test and an I^2 50%, P 0.10, indicated the possibility of significant statistical heterogeneity among the trials, and the random effects model was adopted. When I^2?50%, P ? 0.10, it indicated the possibility of no obvious statistical heterogeneity among the trials, and the fixed effects model was used. In this review, the fail-safe number (Nfs) was used to determine the publication bias. A high value of the Nfs indicated a good robustness of the results, and the publication bias may have no statistically significant influence on the results.

29. * Analysis of subgroups or subsets.

State any planned investigation of ‘subgroups’. Be clear and specific about which type of study or participant will be included in each group or covariate investigated. State the planned analytic approach.

We planned to conduct subgroup analyses for different kinds of outcomes data or characteristics of risk factors if data were available.

Sensitivity analysis was performed when possible to test the robustness of the results.

30. * Type and method of review.

Select the type of review, review method and health area from the lists below.

Type of review

Cost effectiveness
No

Diagnostic
No

Epidemiologic
Yes

Individual patient data (IPD) meta-analysis
No

Intervention
No

Meta-analysis
Yes

Methodology
No

Narrative synthesis
No

Network meta-analysis
No

Pre-clinical
No

Prevention
No

Prognostic
No

Prospective meta-analysis (PMA)
No

Review of reviews
No

Service delivery
No

Synthesis of qualitative studies
No

Systematic review
Yes

Other
No

Health area of the review

Alcohol/substance misuse/abuse
No

Blood and immune system
No

Cancer
No

Cardiovascular
No

Care of the elderly
No

Child health
No

Complementary therapies
No

COVID-19
No

Crime and justice
No

Dental
No

Digestive system

No

Ear, nose and throat
No

Education
No

Endocrine and metabolic disorders
No

Eye disorders
No

General interest
No

Genetics
No

Health inequalities/health equity
No

Infections and infestations
No

International development
No

Mental health and behavioural conditions
No

Musculoskeletal
No

Neurological
No

Nursing
No

Obstetrics and gynaecology
No

Oral health
No

Palliative care
No

Perioperative care
No

Physiotherapy
No

Pregnancy and childbirth
Yes

Public health (including social determinants of health)
No

Rehabilitation
No

Respiratory disorders
No

Service delivery
No

Skin disorders
No

Social care
No

Surgery
No

Tropical Medicine
No

Urological
No

Wounds, injuries and accidents
No

Violence and abuse
No

31. Language.

Select each language individually to add it to the list below, use the bin icon to remove any added in error.
English

There is an English language summary.

32. * Country.

Select the country in which the review is being carried out. For multi-national collaborations select all the countries involved.

China

33. Other registration details.

Name any other organisation where the systematic review title or protocol is registered (e.g. Campbell, or The Joanna Briggs Institute) together with any unique identification number assigned by them. If extracted data will be stored and made available through a repository such as the Systematic Review Data Repository (SRDR), details and a link should be included here. If none, leave blank.

34. Reference and/or URL for published protocol.

If the protocol for this review is published provide details (authors, title and journal details, preferably in Vancouver format)

Add web link to the published protocol.

Or, upload your published protocol here in pdf format. Note that the upload will be publicly accessible.

Yes I give permission for this file to be made publicly available

Please note that the information required in the PROSPERO registration form must be completed in full even if access to a protocol is given.

35. Dissemination plans.

Do you intend to publish the review on completion?

Yes

Give brief details of plans for communicating review findings.?

36. Keywords.

Give words or phrases that best describe the review. Separate keywords with a semicolon or new line. Keywords help PROSPERO users find your review (keywords do not appear in the public record but are included in searches). Be as specific and precise as possible. Avoid acronyms and abbreviations unless these are in wide use.

37. Details of any existing review of the same topic by the same authors.

If you are registering an update of an existing review give details of the earlier versions and include a full bibliographic reference, if available.

38. * Current review status.

Update review status when the review is completed and when it is published.New registrations must be ongoing so this field is not editable for initial submission.
Please provide anticipated publication date

Review_Ongoing

39. Any additional information.

Provide any other information relevant to the registration of this review.

40. Details of final report/publication(s) or preprints if available.

Leave empty until publication details are available OR you have a link to a preprint (NOTE: this field is not editable for initial submission). List authors, title and journal details preferably in Vancouver format.

Give the link to the published review or preprint.
