# Supplementary material for: Risk factors for lactation mastitis in China: A systematic review and meta-analysis
Source: PLoS One. 2021 May 13;16(5):e0251182. doi: 10.1371/journal.pone.0251182 (PMC8118550; doi:10.1371/journal.pone.0251182)
Supplement: S3 File — (DOCX) [file pone.0251182.s003.docx]

**Search** **strategy:**

T The following search terms were used: (mastitis or acute mastitis) and (risk factor or risk factors or influence factors or factor analysis) and (Chinese or China). A total of 265 related articles were obtained from 6 databases.

**December 5, 2020**

| **Database** | **Results** |
| --- | --- |
| **PubMed** | 5 |
| **Web of science** | 7 |
| **China National Knowledge Infrastructure (CNKI)** | 78 |
| **China Science Technology Journal Database (VIP)** | 50 |
| **Wan fang Database** | 76 |
| **Chinese Biomedical Literature Database (SinoMed)** | 49 |

Search terms in PubMed:

#1 Mastitis [MeSH Terms] OR acute mastitis

#2 risk factor [MeSH Terms] OR risk factors OR influence factors OR factor analysis

#3 Chinese OR China

#4 #1AND#2AND#3

Search strategy:

#1 Search: (mastitis [MeSH Terms]) OR (acute mastitis[Title/Abstract])

**4,726 hits**

#2 Search: ((((risk factor [MeSH Terms])) OR (risk factors[Title/Abstract])) OR (influence factors[Title/Abstract])) OR (factor analysis[Title/Abstract])

**1,126,****949 hits**

#3 Search: (Chinese) OR (China)

**2,163,003 hits**

#4 Search: (((mastitis[MeSH Terms]) OR (acute mastitis[Title/Abstract])) AND (((((risk factor[MeSH Terms]) ) OR (risk factors[Title/Abstract])) OR (influence factors[Title/Abstract])) OR (factor analysis[Title/Abstract]))) AND ((Chinese) OR (China))

**5 hits**
