# Supplementary material for: Risk factors for lactation mastitis in China: A systematic review and meta-analysis
Source: PLoS One. 2021 May 13;16(5):e0251182. doi: 10.1371/journal.pone.0251182 (PMC8118550; doi:10.1371/journal.pone.0251182)
Supplement: S4 File — (DOCX) [file pone.0251182.s004.docx]

Summary of Abbreviations in text.

CI: confidence intervals; RCT: randomized controlled trial; OR: odds ratio; REM: random effect model; FIX: fixed effect model; Nfs: fail-safe number; case: Case group, control: Control group, NR: Not reported; NA: not available; LM: Lactation mastitis; WHO: the World Health Organization; PAR: the population attributable risks percent; NOS: the criteria of Newcastle-Ottawa Scale.
