# Supplementary material for: Risk factors for lactation mastitis in China: A systematic review and meta-analysis
Source: PLoS One. 2021 May 13;16(5):e0251182. doi: 10.1371/journal.pone.0251182 (PMC8118550; doi:10.1371/journal.pone.0251182)
Supplement: S1 Table — (DOCX) [file pone.0251182.s005.docx]

**S1 Table. Study quality of case-control studies**

| **Study ID** | **Is the case definition adequate?** | **Representativeness of the cases** | **Selection of controls** | **Definition of controls** | **Comparability of cases and controls on the basis of the design or analysis** | **Ascertainment of exposure** | **Same method of ascertainment for cases and controls** | **Non-response rate** | **Total Scores** |
| --- | --- | --- | --- | --- | --- | --- | --- | --- | --- |
| **Zhong HY 2018[21]** | **1** | **1** | **1** | **1** | **0** | **1** | **1** | **0** | **6** |
| **He XP2013[22]** | **1** | **1** | **1** | **1** | **2** | **1** | **1** | **0** | **8** |
| **Pu YN2017[23]** | **1** | **1** | **1** | **1** | **0** | **1** | **1** | **0** | **6** |
| **Li JX2019[24]** | **1** | **1** | **1** | **1** | **0** | **1** | **1** | **0** | **6** |
| **Wang HM2016[25]** | **1** | **1** | **1** | **1** | **2** | **1** | **1** | **0** | **8** |
| **ChengMH2014[26]** | **1** | **1** | **1** | **1** | **0** | **1** | **1** | **0** | **6** |
| **Zhai HL2017[27]** | **1** | **1** | **1** | **1** | **2** | **0** | **1** | **0** | **7** |
| **Gao X2015[28]** | **1** | **1** | **1** | **0** | **2** | **1** | **1** | **0** | **7** |
| **Chen XG2016[29]** | **1** | **1** | **1** | **1** | **0** | **1** | **1** | **0** | **6** |
| **Yin YS2020[30]** | **1** | **1** | **1** | **1** | **2** | **1** | **1** | **0** | **8** |
| **HuXC2020[31]** | **1** | **1** | **1** | **1** | **0** | **1** | **1** | **0** | **6** |
