# Supplementary material for: Risk factors for lactation mastitis in China: A systematic review and meta-analysis
Source: PLoS One. 2021 May 13;16(5):e0251182. doi: 10.1371/journal.pone.0251182 (PMC8118550; doi:10.1371/journal.pone.0251182)
Supplement: S2 Table — (DOCX) [file pone.0251182.s006.docx]

**S2 Table. Study quality of cohort studies**

| **Study ID** | **Representativeness of the exposed cohort** | **Selection of the non-exposed cohort** | **Ascertainment of exposure** | **Demonstration that outcome of interest was not present at start of study** | **Comparability of cohorts** | **Assessment of outcome** | **Was follow-up long enough for outcomes to occur** | **Adequacy of follow-up of cohorts** | **Total score** |
| --- | --- | --- | --- | --- | --- | --- | --- | --- | --- |
| **Li Tang2014[20]** | **1** | **1** | **1** | **1** | **2** | **1** | **1** | **0** | **8** |
