# Supplementary material for: Risk factors for lactation mastitis in China: A systematic review and meta-analysis
Source: PLoS One. 2021 May 13;16(5):e0251182. doi: 10.1371/journal.pone.0251182 (PMC8118550; doi:10.1371/journal.pone.0251182)
Supplement: S3 Table — (DOCX) [file pone.0251182.s007.docx]

**S3 Table.** **Study quality of cross-sectional studies.**

| **Study ID** | **Representativeness of the sample** | **Sample size** | **Non-respondents** | **Ascertainment of the exposure (risk factor)** | **Comparability** | **Assessment of the outcome** | **Statistical test** | **Total score** |
| --- | --- | --- | --- | --- | --- | --- | --- | --- |
| **Xia HL2011[32]** | **1** | **0** | **1** | **2** | **1** | **2** | **1** | **8** |
| **Wang XL2018[33]** | **1** | **0** | **1** | **1** | **1** | **1** | **1** | **6** |
